# Alpha 1-Antichymotrypsin, an Inflammatory Protein Overexpressed in the Brains of Patients with Alzheimer's Disease, Induces Tau Hyperphosphorylation through c-Jun N-Terminal Kinase Activation

**DOI:** 10.1155/2013/606083

**Published:** 2013-09-23

**Authors:** Ethika Tyagi, Tina Fiorelli, Michelle Norden, Jaya Padmanabhan

**Affiliations:** ^1^Department of Molecular Medicine, University of South Florida, 12901 Bruce B. Downs Boulevard, Tampa, FL 33612, USA; ^2^USF Health Byrd Alzheimer's Institute, University of South Florida, 4001 E. Fletcher Avenue, Tampa, FL 33613, USA

## Abstract

The association of inflammatory proteins with neuritic plaques in the brains of Alzheimer's disease (AD) patients has led to the hypothesis that inflammation plays a pivotal role in the development of pathology in AD. Earlier studies have shown that alpha 1-antichymotrypsin (ACT) enhances amyloid beta fibrillization and accelerated plaque formation in APP transgenic mice. Later studies from our laboratory have shown that purified ACT induces tau hyperphosphorylation and degeneration in neurons. In order to understand the mechanisms by which inflammatory proteins enhance tau hyperphosphorylation, we injected interleukin-1**β** (IL-1**β**) intracerebroventricularly into mice expressing human ACT, human tau, or both transgenes. It was found that the hyperphosphorylation of tau in ACT and ACT/htau mice after IL-1**β** injection correlated with increased phosphorylation of c-Jun N-terminal kinase (JNK). We verified the involvement of JNK in ACT-induced tau phosphorylation by utilizing JNK inhibitors in cultured primary neurons treated with ACT, and we found that the inhibitor showed complete prevention of ACT-induced tau phosphorylation. These results indicate that JNK is one of the major kinases involved in the ACT-mediated tau hyperphosphorylation and suggest that inhibitors of this kinase may protect against inflammation-induced tau hyperphosphorylation and neurodegeneration associated with AD.

## 1. Introduction

Alpha-1-antichymotrypsin (ACT), also called SERPINA3 [1], is a member of the serine protease inhibitor (serpin) family of acute phase proteins. Although ACT is predominantly produced in the liver, it is also synthesized in the brain, mainly by astrocytes [2]. Elevated levels of ACT are found in the brain [3, 4], serum and cerebrospinal fluid (CSF) [5] of AD patients, and high levels of ACT in plasma is associated with cognitive decline in elderly subjects [6]. This suggests that ACT may serve as a biomarker for early diagnosis of the disease. Studies in transgenic mouse models of AD have shown that expression of a human ACT gene accelerates amyloid plaque formation [7, 8] and cognitive decline [9] confirming the role of ACT in AD pathophysiology. The data from *in vitro* studies suggest that ACT binds A*β* peptide and affects the rate of amyloid fibril formation [10–13], possibly causing the accelerated A*β* aggregation and plaque formation observed *in vivo*.

Various cytokines and growth factors such as interleukin 1 (IL-1), IL-6, tumor necrosis factor alpha (TNF*α*), oncostatin M, and leukemia inhibitory factor have been shown to regulate expression of acute phase proteins such as ACT [14, 15]. IL-1*β*, for example, has been shown to induce expression of ACT in human astrocytoma U373-MG cells [16]. It has been reported that expression of ACT in astrocytes is induced most potently by oncostatin M and IL-1, while TNF-*α* has a modest effect [17]. IL-1 is a pluripotent and proinflammatory molecule that affects expression of other inflammatory cytokines and inflammation associated proteins, thus, amplifying immune and inflammatory responses [18]. IL-1*β* has been reported to accelerate hyperphosphorylation of tau in cultured cortical neurons, indicating that it may play a pivotal role in the progression of AD-related pathology [19], and IL-1*β* also has been shown to enhance ACT expression in mice [20]. For these reasons, we selected IL-1*β* as a tool to accelerate the effect of ACT in transgenic mice expressing hTau.

Hyperphosphorylation and accumulation of tau leading to formation of neurofibrillary tangles (NFT) in neurons and tau aggregation in glial cells are the main pathological hallmarks of AD as well as other tauopathies. In a normal brain, tau binds to and stabilizes the microtubule cytoskeleton, whereas, in AD brain, tau hyperphosphorylation prevents its association with microtubule leading to microtubule destabilization and consequent cytoskeletal dysfunction, NFT formation and neurodegeneration [21, 22]. Previous reports from our laboratory have shown that purified ACT induces tau phosphorylation and apoptosis in primary mouse and human neurons [23]. The findings that ACT is overexpressed in astrocytes surrounding plaques in Alzheimer's disease brain and that it enhances A*β* oligomerization and tau hyperphosphorylation, suggest that it may play a role in tangle formation in the AD brain.

Here, we examined the mechanism of ACT-induced tau phosphorylation in transgenic animals intracerebroventricularly injected with IL-1*β*. Tau is phosphorylated at multiple sites by several kinases. Increased expression of kinases, including stress-activated protein kinase (SAPK)/c-Jun N-terminal kinase (JNK), and p38 kinase have been found in brain homogenates from tauopathy patients [24]. Enhanced activity of SAPK/JNK has been observed specifically in the neurons and glial cells containing hyperphosphorylated tau, as well as in dystrophic neurites surrounding the senile plaques in AD. The JNK pathway is of particular interest as activation of JNK can lead to apoptosis and therefore may underlie the neurodegeneration seen in AD. Also, this pathway has been demonstrated to be activated by A*β* [25] and causes hyperphosphorylation of tau [26, 27] suggesting that JNK may lie at an intersection between the two major pathological hallmarks of AD. 

The JNK signaling pathway can be activated by a number of stress factors, including oxidative stress and proinflammatory cytokines [28]. The activation of JNK can induce abnormal phosphorylation of proteins that are not targets of JNK under normal conditions [29]. Studies using cell culture models [30] have shown that JNK induces tau hyperphosphorylation leading to caspase activation and tau cleavage. Thus, in cultured neurons JNK activation may lead to activation of apoptotic pathways and neurodegeneration [29]. This suggested that analysis of ACT's effect on JNK activation and tau hyperphosphorylation in neurons may provide us with important information on the mechanisms by which this inflammatory protein affects development of pathology in AD. 

The mice expressing human ACT that we used in our studies expressed very low levels of ACT. Therefore, in order to study the role of ACT and JNK on tau phosphorylation, we examined cultured cortical neurons treated with ACT as well as transgenic mice expressing ACT and tau after intracranial injection with IL-1*β*. The results from these studies are discussed below.

## 2. Material and Methods

The tissue culture reagents and electrophoresis supplies used in this study were purchased from Gibco/Invitrogen, Carlsbad, CA. *α*1-ACT (ACT) was from RDI Division of Fitzgerald Industries, Intl. (Concord MA), Poly-D-lysine (PDL) was from Sigma (St Louis, MO), recombinant IL-1*β* was purchased from R&D Systems (Minneapolis, MN); and JNK inhibitor (SP600125) was purchased from Calbiochem, p-Thr231 phospho-tau antibody was from AnaSpec Inc. (CA), and p-Ser262 phospho-specific tau antibody was from Biosource International (Camarillo, CA). Antibodies to PHF-1 and P-Ser202-tau (CP13) were kind gifts from Dr. Peter Davies, Albert Einstein College of Medicine, NY. Alexa Fluor 488 and 594 secondary antibodies were purchased from Invitrogen/Molecular Probes. Enhanced chemiluminescence (ECL) reagent was from Pierce Biotechnology Inc. (Rockford, IL). The nitrocellulose membrane was from Schleicher and Schuell (Keene, NH).

### 2.1. Transgenic Mice

Studies involving animals were done in strict accordance with the rules and regulations set forth by the University of South Florida's Institutional Animal Care and Use Committee (IACUC). The care for the animals was provided by the well-established animal care facility at the University of South Florida (USF), which is accredited by the American Association of Laboratory Animal Care (AALAC). We used approximately 9 months old male and female mice in our studies. Construction of the ACT mice was described previously [7]. The human tau (hTau) mice [31] were purchased from Jackson laboratories. These mice express human tau on a mouse tau (mTau) null background. We bred these mice with ACT mice to generate the different transgenic lines used in this study. In the current study we used mTau, ACT/mTau, and ACT/mTau/hTau mice to examine the changes in tau phosphorylation induced by IL-1*β*. 

### 2.2. Genotype Analysis of hTau and ACT Transgenic Mice

Tail snips taken from the hTau mice (Jackson Laboratory, strain B6Cg Mapt^tml(GFP)Klt^Tg^(MAPT)^ 8cPdav/J, stock number 005491) were digested using DNeasy Blood and Tissue kit (Qiagen 69506). Real-time PCR analysis was done using the following probes and primers. Probes (hTau: 5′-/56-RoxN/ATG CAC CAA GAC CAA GAG GGT GAC A/3BHQ_2/-3′; mut mTau: 5′/5Cy5/AGC ACG ACT TCT TCA AGT CCG CCA T/BHQ_2/-3′; mTau: 5′-/56-FAM/ACC CTC GCC AGG AGT TTG ACA CAA T/3BHQ_1/-3′) and primers (hTau: F5′-ACT TTG AAC CAG GAT GGC TGA GCC C-3′, R5′-CTG TGC ATG GCT GTC CAC TAA CCT T-3′; mut mTau: F5′-AAG TTC ATC TGC ACC ACC-3′, R5′-TCC TTG AAG AAG ATG GTG CG-3′; and mTau: F5′-CTC AGC ATC CCA CCT GTA AC-3′, F5′-CCA GTT GTG TAT GTC CAC CC-3′) (IDT). Primers, probes, samples, and iQ Multiplex Powermix (Bio-Rad 172-5848) were loaded into 96-well PCR plates (Bio-Rad 2239441) and amplified using a Bio-Rad i-cycler, under standard PCR conditions, to detect products in respective wavelengths. Genotyping for ACT was performed as above in a separate reaction, using primers F5′-CAG AGC CAG GAG AGG TAA CAT TCT-3′ and R5′-GCA GAT TTA GTC CAA CCC GTT CCT-3′ and probe 5′-/5TexRd-XN/ACT CTA CTC CAG GGA ATT GGT CGA CT/3BHQ_2/-3′.

### 2.3. Intracerebroventricular (ICV) Injection of IL-1*β*

Animals were anesthetized with 1-2% isoflurane, shaved, scrubbed with 10% betadine solution at the site of incision and placed into a dual arm stereotaxic frame (Kopf Instruments, Tujunga, CA). A small (1-2 cm caudal to rostral) incision was made, exposing the skull. Bilateral ICV injection of IL-1*β* (1 ng/10 *μ*L), dissolved in artificial CSF, was slowly injected (5 *μ*L on each site) using a Hamilton microsyringe. The coordinates used for the injection are as follows: from Bregma −0.2 mm anterior-posterior, ±1.0 mm medial lateral and to a depth of 2.2–2.5 mm. The syringe was inserted and left for 5 minutes before solution administration to avoid tissue damage and fluid backflow. Similarly, the needle was left at the site for 5 minutes after injection, before withdrawal. Mice injected with same volume of artificial CSF (aCSF) served as control group. Depth of anesthesia was determined by toe pinch and corneal reflex periodically during the procedure.

### 2.4. Tissue Collection and Sample Preparation

Mice were anesthetized using Nembutal and perfused with saline solution 24 hours after IL-1*β* injection. The hippocampus (HP), cerebral cortex (CC), and striatum (STR) were dissected out and used for extraction of proteins. The brain regions were homogenized in RIPA lysis buffer containing 100 mM Tris, pH 8.0, 150 mM NaCl, 1% NP-40, 0.5% deoxycholate, 0.1% SDS, 1 mM Na_3_VO_4_, 1 mM NaF, 1 mM PMSF, and protease inhibitors (1 complete-mini tablet/10 mL lysis buffer, Roche). Samples were centrifuged at 14,000 rpm, for 30 min at 4°C, and equal amounts of proteins were used for PAGE and western blot analysis.

### 2.5. Cortical Neuronal Cultures

Timed pregnant C57BL/6 mice were obtained from Harlan. Cortical neurons were cultured from pups taken at embryonic day 18 as described previously [32]. Briefly, the animals were anesthetized with Nembutal and the embryos were dissected out, brains removed, and triturated in PBS. The dissociated cortex was centrifuged, and the cells resuspended in neurobasal media with B27 supplement and plated onto polylysine-coated culture plates. Nonneuronal cells were removed by treatment with fluorodeoxyuridine (FDU) after 18 hours of culturing. The cells were replenished with 50% of fresh media every third day. The experiments were done using 1-week-old cultures. ACT was reconstituted according to the manufacturer's protocol and used at a concentration of 1 mg/mL in the culture media, an ACT level similar to that found in AD brain [4, 33].

### 2.6. Immunocytochemistry

Cortical neurons were plated on 8-chamber slides precoated with poly D-lysine for immunocytochemical analysis. Cells were cultured as indicated above for 1 week. ACT was added to the wells at a concentration of 1 mg/mL. At the end of the treatment, cells were fixed with 4% paraformaldehyde for 10 minutes, washed with PBS, and incubated for 1 hour at room temperature with 10% normal goat serum (NGS) in Tris-buffered saline containing 0.2% Triton-X-100 (TBST) to inhibit nonspecific binding. Primary antibodies (PHF-1, p-Ser202, p-Thr231, and p-Ser262 antibodies specific for P-Tau) diluted in 1% bovine serum albumin (BSA)/TBST were added to the cells and incubated overnight at 4°C in a humidified chamber. Cells were washed several times with PBS and incubated with anti-mouse and anti-rabbit secondary antibodies (Alexa Fluor 488 and 594, resp.) for 1 hour at room temperature. Cells were counterstained with Hoechst (1 *μ*g/mL in PBS) to visualize nuclei. Slides were washed in PBS and mounted using aquamount. The staining was visualized and analyzed under a Zeiss fluorescence microscope using the AxioVision Rel. 4.8 software. 

### 2.7. Western Blot Analysis

For analysis of brain samples, equal amounts of proteins from the extracts made in RIPA-lysis buffer were boiled with Tris-glycine gel loading buffer and separated on a 10% Tris-glycine gel. The proteins were electroblotted to a nitrocellulose membrane. Nonspecific binding was blocked by incubation with 5% nonfat dry milk in TBS for 1 hour at room temperature and incubated overnight at 4°C with primary antibodies diluted in 3% BSA/TBS. The blots were washed thoroughly with PBS containing 0.05% Tween-20 and incubated with horse radish peroxidase-conjugated secondary antibodies diluted in blocking buffer for 2 hours at room temperature. After further washes the blots were exposed to super signal ECL reagent, and the signals were detected using autoradiography films.

### 2.8. Statistical Analysis

**Figure 1:**
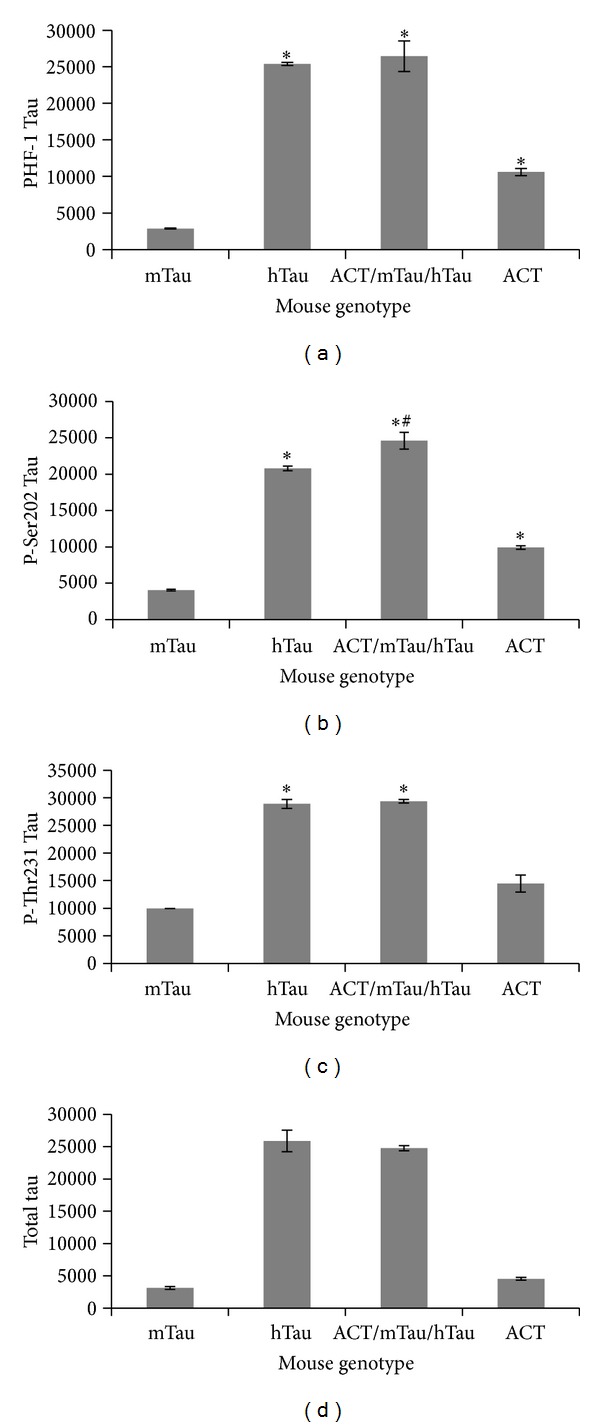
Tau hyperphosphorylation in ACT expressing mice: cortex and hippocampal regions from normal nontransgenic (mTau), ACT expressing (ACT/mTau), human tau expressing (hTau), and both ACT and hTau expressing (ACT/mTau/hTau) mice were analyzed for changes in phosphorylation of tau at PHF-1 (a), p-Ser202 (b), pThr231 (c) specific epitopes, and total tau (d) using the corresponding P-tau or the TG5 total tau antibody by western blot and quantified using the Image J image analysis software. The data shows significant increase in P-tau levels in ACT and hTau as well as the double transgenic ACT/hTau mice (*P < 0.05). Only P-Ser202 tau showed significant change in double transgenic mice compared to mice expressing hTau alone (^#^ *P* < 0.05).

**Figure 2:**
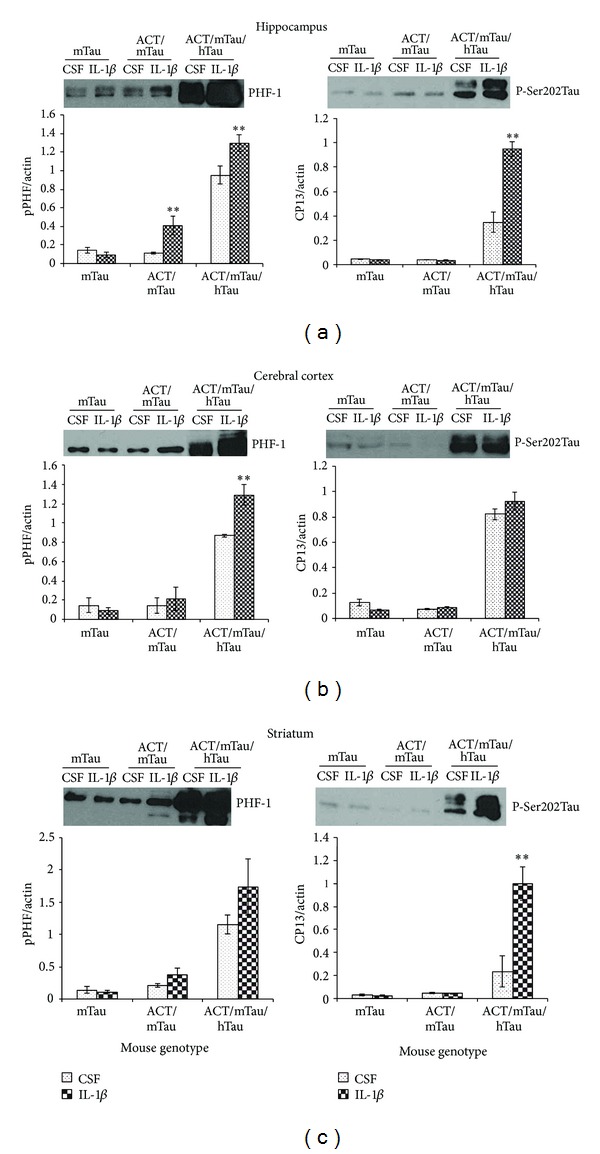
Inflammatory proteins induce tau hyperphosphorylation in AD-associated regions in the brain: PHF-1 and p-Ser202 Tau phosphorylation in different brain regions—hippocampus (a), cerebral cortex (b), and striatum (c) from control mice (mTau), and mouse expressing ACT/mTau and ACT/mTau/hTau transgenes injected with CSF and IL-1*β* (1 ng/10 *μ*L) *P < 0.05, **P < 0.01 (calculated from aCSF *versus* IL-1*β* treated group). Values were normalized to actin levels. The blot above each bar graph shows a representative western blot showing corresponding P-tau in the samples from different mice. The data was collected from four independent mice from each genotype group.

**Figure 3:**
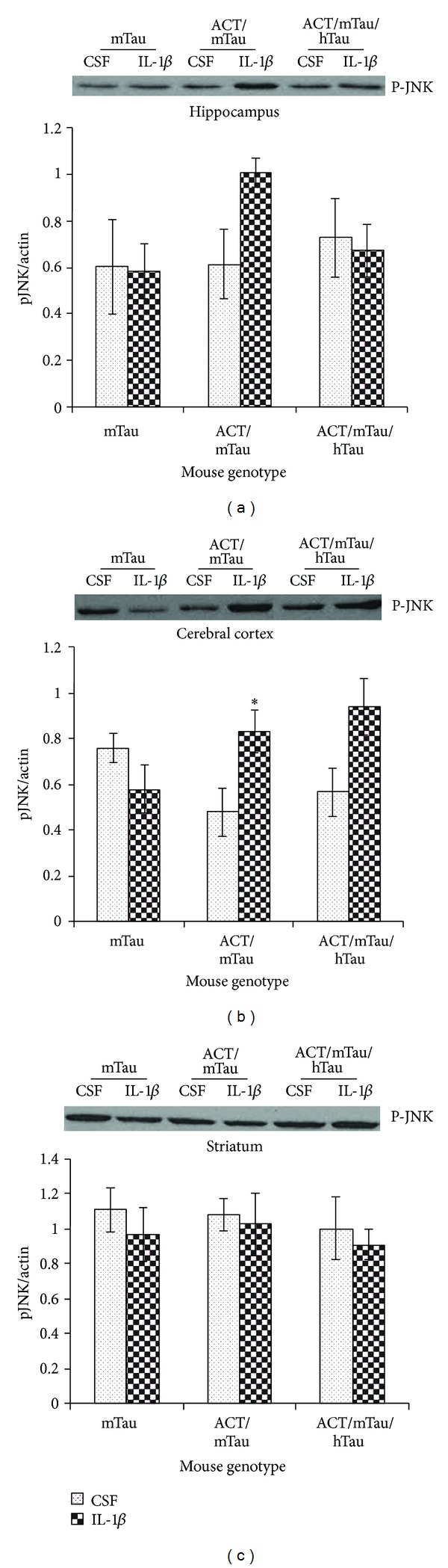
Inflammatory proteins induce tau phosphorylation through JNK activation: JNK phosphorylation in different brain regions—hippocampus (a), cerebral cortex (b), and striatum (c), in mTau, ACT/mTau and ACT/mTau/hTau mice injected with aCSF and IL-1*β* (1 ng/10 *μ*L) *P < 0.05 (aCSF *versus* IL-1*β* treated group). Values are normalized to actin levels. The blot above each bar graph shows a representative western blot showing corresponding P-tau in the samples from different mice. The data was collected from four independent mice from each genotype group.

The data shown in Figures 2 and 3 were analyzed using a two-way analysis of variance (ANOVA), with genotype and treatment as the independent variables. Post-hoc analysis was performed using a Fisher's protected Least Significant Difference test to compare IL-1*β* treated animals with aCSF-injected animals within each genotype. Data shown in Figure 1 was analyzed using Student's *t*-test. A *P* value of <0.05 was considered statistically significant.

## 3. Results

### 3.1. Increased Tau Phosphorylation in Mice Expressing the Human ACT Gene Alone and in Combination with Human Tau

The association of ACT with tauopathy and tau hyperphosphorylation is quite new [23]. In order to determine the correlation between tau hyperphosphorylation and ACT expression *in vivo*, we first examined the level of tau phosphorylation in ACT and ACT/hTau mice. Cortical brain regions were extracted with RIPA lysis buffer, and tau hyperphosphorylation analyzed using specific P-tau antibodies. As expected the hTau mice and the doubly transgenic ACT/hTau mice showed significantly higher levels of total tau and P-tau in the brain extracts compared to the normal control and ACT only mice (Figure 1). Similar to the results we observed in our previous studies [23], ACT expression in the mice was associated with an increase in PHF-1, P-Ser202, and P-Thr231 P-tau epitopes (Figures 1(a)–1(c)). The levels of P-Ser202-tau (Figure 1(b)) were significantly increased in mice expressing ACT and hTau as well (ACT/mTau/hTau); PHF-1 and P-thr231 tau levels appeared to be enhanced in ACT/mTau/hTau compared to hTau alone but the increase was not significant (Figures 1(a) and 1(c)). It is possible that the high levels of P-tau already present in the hTau mice obscure the changes brought about by ACT expression. The total level of tau seems to be unaffected by ACT expression in the hTau and control mice (Figure 1(d)). These findings that ACT enhances hyperphosphorylation of tau at sites that are specifically phosphorylated in AD suggest that proteins associated with inflammation, in this case ACT, may alter normal tau to tangle prone tau. Further analysis is necessary to determine whether ACT enhances aggregation of tau and tangle formation in these mice.

### 3.2. Intracranial Injection of IL-1*β* Induces Tau Hyperphosphorylation in Specific Regions in the Brain

The ability of IL-1*β* to induce hyperphosphorylation of tau in cortical neurons [19] and the fact that IL-1*β* induces ACT expression [20] encouraged us to explore the effect of IL-1*β* on Tau phosphorylation in nontransgenic (mTau) and transgenic mice expressing ACT (ACT/mTau and ACT/mTau/hTau). Nontransgenic normal mice (mTau) and mice expressing ACT alone or ACT and hTau were intracranially injected with 5 *μ*L (1 ng/10 *μ*L) IL-1*β* dissolved in aCSF. The control mice received the same volume of aCSF injection. Mice were sacrificed 24 hour after injection, and microsurgery was performed to remove hippocampus, cortex, and striatum for analysis of P-tau. We observed a significant induction in PHF-1 specific tau phosphorylation in the samples from hippocampus and cortex but not in striatum after IL-1*β* injection in ACT/mTau/hTau mice (Figures 2(a)–2(c)). The hippocampal region from ACT/mTau mice showed significant increase in PHF-1 while the cortex or striatum did not show any significant change. Analysis of P-Ser202 specific tau phosphorylation showed significant increase only in the hippocampus and striatum from ACT/mTau/hTau mice and not ACT/mTau mice. Cortical region did not show any change in the levels of P-Ser202 tau after IL-1*β* injection. These results suggest that inflammatory proteins play a pivotal role in induction of specific tau hyperphosphorylation and possibly microtubule instability, leading to tangle formation in brain.

### 3.3. IL-1*β* Injection Induces JNK Phosphorylation in Mice Expressing the Human ACT Gene Alone and in Combination with Human Tau

Next, we examined the signaling pathways involved in ACT-induced tau phosphorylation upon stimulation by IL-1*β*. The JNK pathway is of particular interest as activation of JNK can lead to tau phosphorylation and tau cleavage as well as apoptosis in neurons. Therefore, we decided to examine the activation state of JNK after the inflammatory stimulation by IL-1*β* injection in ACT/mTau or ACT/mTau/hTau mice. Brain extracts were prepared from mice after intracranial injection with IL-1*β* or aCSF and JNK phosphorylation analyzed by western blot using a P-JNK antibody. We observed a significant increase in phosphorylation of JNK in the cortex region from ACT/mTau and a slight increase in the ACT/mTau/hTau mice, indicating a possible role for JNK activation in ACT-induced tau phosphorylation (Figure 3(b)). There was no significant change in P-JNK levels in the striatum or hippocampal region from ACT/mTau or ACT/mTau/hTau mice (Figures 3(a) and 3(c)) suggesting that the tau phosphorylation observed in these regions is brought about by kinases other than JNK. Our earlier studies in cultured neurons have shown that ACT-mediated tau hyperphosphorylation is associated with activation of GSK3*αβ* and ERK [23]. Here we found that JNK kinases are activated in response to ACT which in turn induces tau hyperphosphorylation. Although initial *in vivo *analysis did not indicate any activation of GSK3*αβ* or ERK, further analysis is necessary to confirm these results.

### 3.4. Effect of JNK Inhibitor on ACT-Induced Tau Phosphorylation in Primary Cortical Neurons

**Figure 4:**
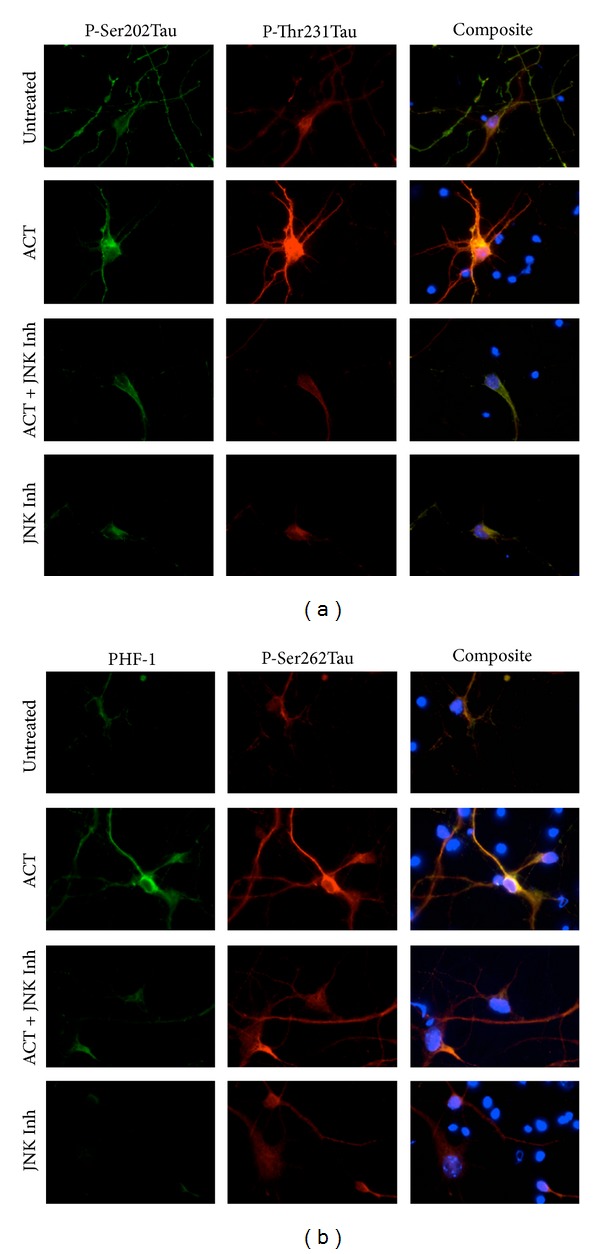
ACT-mediated tau phosphorylation at p-Ser202, p-Thr231, PHF-1, and p-Ser262 in cortical neurons is inhibited by JNK inhibitor: mouse cortical neurons were cultured in 8-chamber slides and preincubated with JNK Inhibitor (10 *μ*M, 1 hour). The cells were then treated with or without 1 mg/mL ACT for 8 hours and probed with (a) p-Ser202 tau monoclonal and p-Thr231 tau polyclonal, (b) PHF-1 tau monoclonal and P-Ser262 tau polyclonal antibodies. Anti-mouse Alexa fluor 488 (green) and anti-rabbit Alexa Flour 594 (red) were used as secondary antibodies, and Hoechst was used to visualize the nuclei. Staining was visualized under a Zeiss fluorescent microscope and analyzed using AxioVision Rel. 4.8 software.

To investigate our hypothesis that ACT enhances tau hyperphosphorylation through a JNK-dependent mechanism, we decided to investigate the effect of JNK inhibitor on tau phosphorylation in primary neurons cultured *in vitro* and treated with ACT. Towards this, we pretreated cortical neurons with 10 *μ*M of JNK inhibitor (SP600125) for 1 hour prior to addition of ACT and continued the incubation for 8 hours in the presence of ACT. The result showed an induction in phosphorylation of tau at different sites, which included PHF-1, p-Ser202, p-Thr231, and p-Ser262, upon ACT treatment (Figures 4(a) and 4(b)). Treatment with JNK inhibitor (SP600125) abolished the ACT-induced tau phosphorylation, indicating the regulatory role of JNK in the process.

## 4. Discussion

The involvement of ACT in AD is well documented, and its role in A*β* oligomerization has been established. In the study presented here we sought to elucidate the mechanisms of ACT-induced tau phosphorylation, focusing on the role of JNK kinase, in order to increase our understanding of how this relevant kinase influences tau hyperphosphorylation and development of pathology in AD. Our studies illustrate that ACT by itself or in conjunction with IL-1*β* induces tau phosphorylation and this is, at least in part, mediated by JNK activation. Mixed cultures of human glia from amyloid-prone cortical tissue show activation and expression of IL-1 and ACT, whereas mixed glial cultures from cerebellum failed to show such inflammatory response, suggesting that the regional specificities of amyloid deposition in AD may reflect on basic differences in inflammatory capacity between different brain regions [34]. For this reason, in the present study we decided to choose the brain regions that show pathological modifications in AD and examined them for changes in tau.

The microglia surrounding the plaques in AD brain have been shown to express high levels of the cytokine IL-1*β* and correlate with the extent of the pathology associated with AD [35]. This suggests that the elevated levels of A*β* may be causing an inflammatory response inducing enhanced expression of proteins associated with inflammation such as IL-1*β*, ACT, and complement factors. IL-1*β* has been shown to enhance the expression of ACT in human astrocytes and to increase the translation of amyloid precursor protein [7, 34]. The findings that ACT is associated with Alzheimer's amyloid plaques and is expressed only in regions where there is overexpression of IL-1*β* suggest that this coupled expression may be of significant importance to the pathogenesis of plaques and tangles associated with the disease. We found that IL-1*β* injection in ACT/hTau mice induced phosphorylation of tau, which is paralleled with JNK phosphorylation, suggesting a role for JNK activation in ACT-induced tau phosphorylation. IL-1*β* enhanced PHF-1 specific tau phosphorylation in hippocampus of ACT/mTau mice, but it did not show a significant change in any other specific tau phosphorylation in either cortex or striatum. This might be due to some compensatory mechanisms or to the inadequacy of inflammatory proteins alone to initiate the degeneration in mouse neurons. It is possible that when hTau is overexpressed in these neurons they respond differently to the inflammatory proteins to elicit a profound change in tau phosphorylation. This could be the reason why under normal conditions mice do not usually show tau tangle formation or neurodegeneration without overexpression of a mutant or WT human tau isoform. Our earlier studies have shown enhanced expression of ACT in neurons and glial cells in tauopathy brain samples, which correlated with tangle pathology [23]. Although the sequence of events involved in tangle formation and neurodegeneration are not yet known, the results from the present study suggest that inflammatory processes can exacerbate the tau modifications but is not able to initiate the process by itself in mice.

There are 79 potential phosphorylation sites in the longest isoform of tau, of which over 30 have been shown to be phosphorylated [36]. Numerous kinases can phosphorylate tau, including cyclin-dependent kinases [37], glycogen synthase kinase 3 (GSK3) [38], microtubule affinity regulating kinase (MARK) [39], Ca^2+^/calmodulin-dependent protein kinase II (CaMKII) [40], protein kinase A (PKA) [41], and members of the mitogen activated protein kinase (MAPK) family including ERK2 [39] and c-jun N-terminal kinase (JNK) [26, 27]. Many of these kinases have been proposed as being critical in causing tau hyperphosphorylation in AD and are potential drug targets [42]. Activation of JNK is seen in animal models of AD [43, 44] and has been observed in clinical cases of AD [45]. Phosphorylation of tau *in vivo* can also occur rapidly in response to stresses including hypothermia [46]. JNK proteins are stress activated protein kinases whose activation may be achieved by diverse triggers including environmental factors and activation of specific receptors [47]. An upstream kinase signaling cascade leads to the phosphorylation of JNK that prototypically translocate to the nucleus, where they phosphorylate transcription factors including c-jun, which are involved in transcription of genes involved in a diverse array of cellular processes including cellular growth and apoptosis [48]. JNKs also have extranuclear targets such as Bcl family members [49] and microtubule associated proteins such as tau [26, 27, 50]. Although we have observed an increase in GSK-3*αβ* and ERK activation in neurons treated with ACT [23], we were unable to detect this *in vivo. *It is possible that the complex conditions that induce tau hyperphosphorylation *in vivo *are different from that we have observed *in vitro.* Further studies are in progress examining the changes in other tau kinases that might play a role in the phosphorylation of tau observed *in vivo.*

The stress activated protein kinases of the JNK family are most closely linked with initiating apoptotic events [51], and there is evidence that they phosphorylate tau *in vitro* [27, 50]. Our experiments with neurons demonstrate that ACT-induced tau phosphorylation at known JNK phosphorylation sites, Ser202 and Thr231 [50], are significantly increased. Tau can be phosphorylated at multiple sites by an array of kinases, the sequences of phosphorylation events that lead to pathogenicity in AD have been studied [52], but it remains unclear as to how significant each kinase and phosphorylation event may be in the pathogenic cascade. Phosphorylation of tau by JNK is a relatively early event [52], potentially preceded by the action of GSK3*β* on Thr231 [53]. Our results demonstrate that the JNK inhibitor SP600125 can completely reverse the phosphorylation of tau at Ser202, Thr231, and Ser262, indicating that JNK plays a specific role in phosphorylation of tau at these sites. 

## 5. Conclusion

**Figure 5:**
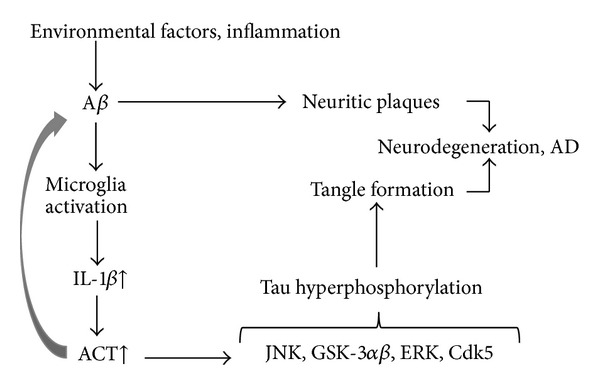
Schematic showing the inflammation-associated mechanisms in tau phosphorylation and tangle formation in brain: environmental factors, injury to brain, or enhanced inflammation induce A*β* generation as well as microglial activation which leads to increased generation of IL-1*β* (cytokine) which in turn has been shown to enhance ACT expression. ACT has been shown to induce tau hyperphosphorylation through activation of GSK-3*αβ*, or ERK (earlier studies) or JNK (data presented here). This may lead to enhanced tangle formation and neurodegeneration. ACT already has been shown to enhance A*β* aggregation and accelerated plaque formation, whether it enhances tau aggregation and tangle formation needs to be determined.

The results from our studies presented here show that the inflammatory protein ACT induces tau hyperphosphorylation through activation of JNK. Earlier *in vitro* studies from our laboratory have shown that ACT enhances tau hyperphosphorylation through activation of GSK-3*αβ* and ERK [23].  Figure 5 shows a schematic depicting the pathway that may be involved in ACT-induced neurodegeneration in AD. Since ACT expression is significantly upregulated in AD brain, we believe that it plays a major role in tangle formation by participating in upregulation of kinases involved in tau hyperphosphorylation in neurons. Thus, these studies suggest that a better understanding of the signaling mechanisms involved in inflammation-mediated tau hyperphosphorylation may lead to more effective anti-inflammatory therapeutic strategies for preventing or treating AD neurodegeneration associated with chronic inflammation.

## Abbreviations

AD: Alzheimer's disease
ACT: Alpha 1-antichymotrypsin
JNK: c-Jun N-terminal Kinase
mTau: Mouse tau
hTau: Human tau.

## References

[1] Silverman GA, Bird PI, Carrell RW (2001). The serpins are an expanding superfamily of structurally similar but functionally diverse proteins. Evolution, mechanism of inhibition, novel functions, and a revised nomenclature. *Journal of Biological Chemistry*.

[2] Kalsheker NA (1996). *α*1-antichymotrypsin. *International Journal of Biochemistry and Cell Biology*.

[3] Licastro F, Mallory M, Hansen LA, Masliah E (1998). Increased levels of *α*-1-antichymotrypsin in brains of patients with Alzheimer’s disease correlate with activated astrocytes and are affected by APOE 4 genotype. *Journal of Neuroimmunology*.

[4] Abraham CR, Selkoe DJ, Potter H (1988). Immunochemical identification of the serine protease inhibitor *α*1-antichymotrypsin in the brain amyloid deposits of Alzheimer’s disease. *Cell*.

[5] Licastro F, Parnetti L, Morini MC (1995). Acute phase reactant *α*1-antichymotrypsin is increased in cerebrospinal fluid and serum of patients with probable Alzheimer disease. *Alzheimer Disease and Associated Disorders*.

[6] Dik MG, Jonker C, Hack CE, Smit JH, Comijs HC, Eikelenboom P (2005). Serum inflammatory proteins and cognitive decline in older persons. *Neurology*.

[7] Nilsson LNG, Bales KR, DiCarlo G (2001). *α*-1-antichymotrypsin promotes *β*-sheet amyloid plaque deposition in a transgenic mouse model of Alzheimer’s disease. *Journal of Neuroscience*.

[8] Mucke L, Yu G-Q, McConlogue L, Rockenstein EM, Abraham CR, Masliah E (2000). Astroglial expression of human *α*1-antichymotrypsin enhances Alzheimer-like pathology in amyloid protein precursor transgenic mice. *American Journal of Pathology*.

[9] Nilsson LNG, Arendash GW, Leighty RE (2004). Cognitive impairment in PDAPP mice depends on ApoE and ACT-catalyzed amyloid formation. *Neurobiology of Aging*.

[10] Ma J, Brewer HB, Potter H (1996). Alzheimer A beta neurotoxicity: promotion by antichymotrypsin, ApoE4; inhibition by A beta-related peptides. *Neurobiology of Aging*.

[11] Eriksson S, Janciauskiene S, Lannfelt L (1995). *α*1-antichymotrypsin regulates Alzheimer *β*-amyloid peptide fibril formation. *Proceedings of the National Academy of Sciences of the United States of America*.

[12] Fraser PE, Nguyen JT, McLachlan DR, Abraham CR, Kirschner DA (1993). *α*1-antichymotrypsin binding to Alzheimer A*β* peptides is sequence specific and induces fibril disaggregation in vitro. *Journal of Neurochemistry*.

[13] Abraham CR, McGraw WT, Slot F, Yamin R (2000). *α* 1-antichymotrypsin inhibits A*β* degradation in vitro and in vivo. *Annals of the New York Academy of Sciences*.

[14] Cichy J, Potempa J, Chawla RK, Travis J (1995). Stimulatory effect of inflammatory cytokines on *α*1-antichymotrypsin expression in human lung-derived epithelial cells. *Journal of Clinical Investigation*.

[15] Cichy J, Potempa J, Chawla RK, Travis J (1995). Regulation of *α*1-antichymotrypsin synthesis in cells of epithelial origin. *FEBS Letters*.

[16] Gitter BD, Boggs LN, May PC, Czilli DL, Carlson CD (2000). Regulation of cytokine secretion and amyloid precursor protein processing by proinflammatory amyloid beta (A*β*). *Annals of the New York Academy of Sciences*.

[17] Machein U, Lieb K, Hüll M, Fiebich BL (1995). IL-1*β* and TNF*α*, but not IL-6, induce *α*1-antichymotrypsin expression in the human astrocytoma cell line U373 MG. *NeuroReport*.

[18] Mrak RE, Griffin WST (2000). Interleukin-1 and the immunogenetics of Alzheimer disease. *Journal of Neuropathology and Experimental Neurology*.

[19] Li Y, Liu L, Barger SW, Griffin WST (2003). Interleukin-1 mediates pathological effects of microglia on tau phosphorylation and on synaptophysin synthesis in cortical neurons through a p38-MAPK pathway. *Journal of Neuroscience*.

[20] Morihara T, Teter B, Yang F (2005). Ibuprofen suppresses interleukin-I*β* induction of pro-amyloidogenic *α*1-antichymotrypsin to ameliorate *β*-amyloid (a*β*) pathology in Alzheimer’s models. *Neuropsychopharmacology*.

[21] Brunden KR, Trojanowski JQ, Lee VM-Y (2009). Advances in tau-focused drug discovery for Alzheimer’s disease and related tauopathies. *Nature Reviews Drug Discovery*.

[22] Lee VM-Y, Goedert M, Trojanowski JQ (2001). Neurodegenerative tauopathies. *Annual Review of Neuroscience*.

[23] Padmanabhan J, Levy M, Dickson DW, Potter H (2006). Alpha1-antichymotrypsin, an inflammatory protein overexpressed in Alzheimer’s disease brain, induces tau phosphorylation in neurons. *Brain*.

[24] Ferrer I (2004). Stress kinases involved in Tau phosphorylation in Alzheimer’s disease, tauopathies and APP transgenic mice. *Neurotoxicity Research*.

[25] Minogue AM, Schmid AW, Fogarty MP (2003). Activation of the c-Jun N-terminal kinase signaling cascade mediates the effect of amyloid-*β* on long term potentiation and cell death in hippocampus. A role for interleukin-1*β*?. *Journal of Biological Chemistry*.

[26] Goedert M, Hasegawa M, Jakes R, Lawler S, Cuenda A, Cohen P (1997). Phosphorylation of microtubule-associated protein tau by stress-activated protein kinases. *FEBS Letters*.

[27] Reynolds CH, Utton MA, Gibb GM, Yates A, Anderton BH (1997). Stress-activated protein kinase/c-Jun N-terminal kinase phosphorylates *τ* protein. *Journal of Neurochemistry*.

[28] Cho S-G, Choi E-J (2002). Apoptotic signaling pathways: caspases and stress-activated protein kinases. *Journal of Biochemistry and Molecular Biology*.

[29] Borsello T, Forloni G (2007). JNK signalling: a possible target to prevent neurodegeneration. *Current Pharmaceutical Design*.

[30] Sahara N, Murayama M, Lee B (2008). Active c-jun N-terminal kinase induces caspase cleavage of tau and additional phosphorylation by GSK-3*β* is required for tau aggregation. *European Journal of Neuroscience*.

[31] Andorfer C, Acker CM, Kress Y, Hof PR, Duff K, Davies P (2005). Cell-cycle reentry and cell death in transgenic mice expressing nonmutant human tau isoforms. *Journal of Neuroscience*.

[32] Park DS, Morris EJ, Padmanabhan J, Shelanski ML, Geller HM, Greene LA (1998). Cyclin-dependent kinases participate in death of neurons evoked by DNA-damaging agents. *Journal of Cell Biology*.

[33] Liebermann J, Schleissner L, Tachiki KH, Kling AS (1995). Serum *α*1-antichymotrypsin level as a marker for Alzheimer-type dementia. *Neurobiology of Aging*.

[34] Das S, Potter H (1995). Expression of the Alzheimer amyloid-promoting factor antichymotrypsin is induced in human astrocytes by IL-1. *Neuron*.

[35] Tuppo EE, Arias HR (2005). The role of inflammation in Alzheimer’s disease. *International Journal of Biochemistry and Cell Biology*.

[36] Buée L, Bussière T, Buée-Scherrer V, Delacourte A, Hof PR (2000). Tau protein isoforms, phosphorylation and role in neurodegenerative disorders. *Brain Research Reviews*.

[37] Baumann K, Mandelkow EM, Biernat J, Piwnica-Worms H, Mandelkow E (1993). Abnormal Alzheimer-like phosphorylation of tau-protein by cyclin-dependent kinases cdk2 and cdk5. *FEBS Letters*.

[38] Hanger DP, Hughes K, Woodgett JR, Brion J-P, Anderton BH (1992). Glycogen synthase kinase-3 induces Alzheimer’s disease-like phosphorylation of tau: generation of paired helical filament epitopes and neuronal localisation of the kinase. *Neuroscience Letters*.

[39] Drewes G, Ebneth A, Preuss U, Mandelkow E-M, Mandelkow E (1997). MARK, a novel family of protein kinases that phosphorylate microtubule-associated proteins and trigger microtubule disruption. *Cell*.

[40] Baudier J, Cole RD (1987). Phosphorylation of tau proteins to a state like that in Alzheimer’s brain is catalyzed by a calcium/calmodulin-dependent kinase and modulated by phospholipids. *Journal of Biological Chemistry*.

[41] Litersky JM, Johnson GVW (1992). Phosphorylation by cAMP-dependent protein kinase inhibits the degradation of tau by calpain. *Journal of Biological Chemistry*.

[42] Mazanetz MP, Fischer PM (2007). Untangling tau hyperphosphorylation in drug design for neurodegenerative diseases. *Nature Reviews Drug Discovery*.

[43] Savage MJ, Lin Y-G, Ciallella JR, Flood DG, Scott RW (2002). Activation of c-Jun N-terminal kinase and p38 in an Alzheimer’s disease model is associated with amyloid deposition. *Journal of Neuroscience*.

[44] Puig B, Gómez-Isla T, Ribé E (2004). Expression of stress-activated kinases c-Jun N-terminal kinase (SAPK/JNK-P) and p38 kinase (p38-P), and tau hyperphosphorylation in neurites surrounding *β*A plaques in APP Tg2576 mice. *Neuropathology and Applied Neurobiology*.

[45] Zhu X, Raina AK, Rottkamp CA (2001). Activation and redistribution of c-Jun N-terminal kinase/stress activated protein kinase in degenerating neurons in Alzheimer’s disease. *Journal of Neurochemistry*.

[46] Planel E, Richter KEG, Nolan CE (2007). Anesthesia leads to tau hyperphosphorylation through inhibition of phosphatase activity by hypothermia. *Journal of Neuroscience*.

[47] Pulverer BJ, Kyriakis JM, Avruch J, Nikolakaki E, Woodgett JR (1991). Phosphorylation of c-jun mediated by MAP kinases. *Nature*.

[48] Bohmann D, Bos TJ, Admon A, Nishimura T, Vogt PK, Tjian R (1987). Human proto-oncogene c-jun encodes a DNA binding protein with structural and functional properties of transcription factor AP-1. *Science*.

[49] Deng X, Xiao L, Lang W, Gao F, Ruvolo P, May WS (2001). Novel role for JNK as a stress-activated Bcl2 kinase. *Journal of Biological Chemistry*.

[50] Yoshida H, Hastie CJ, McLauchlan H, Cohen P, Goedert M (2004). Phosphorylation of microtubule-associated protein tau by isoforms of c-Jun N-terminal kinase (JNK). *Journal of Neurochemistry*.

[51] Davis RJ (2000). Signal transduction by the JNK group of MAP kinases. *Cell*.

[52] Luna-Muñoz J, Chávez-Macías L, García-Sierra F, Mena R (2007). Earliest stages of tau conformational changes are related to the appearance of a sequence of specific phospho-dependent tau epitopes in Alzheimer’s disease. *Journal of Alzheimer’s Disease*.

[53] Lin Y-T, Cheng J-T, Liang L-C, Ko C-Y, Lo Y-K, Lu P-J (2007). The binding and phosphorylation of Thr231 is critical for Tau’s hyperphosphorylation and functional regulation by glycogen synthase kinase 3*β*. *Journal of Neurochemistry*.

